# A bibliometric analysis of microbial forensics from 1984 to 2022: progress and research trends

**DOI:** 10.3389/fmicb.2023.1186372

**Published:** 2023-05-16

**Authors:** Xiangnan Guo, Liya Gu, Yue Luo, Shuangshuang Wang, Haibo Luo, Feng Song

**Affiliations:** Department of Forensic Genetics, West China School of Basic Medical Sciences & Forensic Medicine, Sichuan University, Chengdu, China

**Keywords:** microbial forensics, forensic science, bibliometric, progress, research trend

## Abstract

Microbial forensics is a rapidly evolving discipline that has gained significant momentum in recent years. The study evaluated relevant results over the last four decades from 1984 to 2022 all over the world, aiming to analyze the growing trends and research orientations of microbial forensics. Using “microbial forensics” as the search topic in the Web of Science Core Collection, the systematic retrieval identified 579 documents relevant to the field and draw many statistical tables and maps to make the retrieval results visible. According to further bibliometric analysis, there are an increasing number of publications related to microbial forensics from the overall trend, with the highest number of publications recorded in 2021. In terms of the total number of articles, the USA and China were both the leading contributors to the field among 40 countries. The field has developed rapidly in recent years based on the development of next-generation sequencing. Over the course of its development, there are rich keywords in the research of scholars, which focus on diversity and identification. Moreover, despite the early hot topic being PCR (the use of PCR to probe microorganisms), in recent years, the topics, markers, and the potential application of microorganisms in forensic practice have become hot, which also indicates the future research directions of microbial forensic.

## 1. Introduction

Microbial forensics is the branch of microbiology, which can judge the sources from the organism of microbiology by detecting their various features such as the distribution of microorganisms' number and species to provide legal support. This discipline has a long history dating back to the late 19th century, when microorganisms were used as evidence to determine the cause of death of humans and animals.

However, on account of technological limitations, research relevant to microbial forensics made slow progress (Metcalf et al., 2017). Afterward, with the rise of bioterrorism, particularly in 2001, the anthrax letter attacks generated great terror in the public, and thus, there needs a microbial forensics system applied to analyze evidence from a bioterrorism act, biocrime, or inadvertent microorganism/toxin release for attribution purposes (Budowle et al., 2003; Robinson et al., 2020). Consequently, microbial forensics was first proposed as a discipline, and the forensic value of microbiology became apparent (Budowle et al., 2003; Kuiper, 2016). In recent years, with the dramatical drop in the cost of DNA sequencing and the development of biotechnology, the field of forensic microbiology grows rapidly (Lehman, 2012; Metcalf et al., 2017).

Nowadays, the scope of microbial forensics has expanded beyond its original focus on bioterrorism threats. For instance, the soil microbiome began to use as valuable evidence to localize the origin of the item associated with a crime by testing the microbial DNA in it (Heath and Saunders, 2006; Robinson et al., 2020). Apart from that, after studying the evolution of microbial communities during the decomposition of corpses and understanding the diversified microorganism characterized in different scenarios, microbial forensics could presume postmortem interval (PMI) and the cause of death (Uchiyama et al., 2012; Metcalf et al., 2016, 2017). Additionally, based on the study of the composition of microbial communities in different human habitats, the diverse characteristics of microbial communities have become the basis for individual identification in forensic practice (Gilbert, 2015; Schmedes et al., 2017; Williams and Gibson, 2019).

Bibliometric analysis is a statistical method that allows for a thorough analysis of publications and trends in a particular scientific field and identifies the most influenced countries, authors, assessing the directions of the current research. Considering the specific development process of microbial forensics, although it has entered a stage of rapid development, there is relatively few research in this field compared with other forensic disciplines. Therefore, bibliometric analysis is needed to describe the overall situation of microbial forensics. This review would conduct a bibliometric analysis associated with the topic of microbial forensics for the period between 1984 and 2022 by using the Web of Science Core Collection, which would provide a systemic overview of the development rate and research outputs in the field of microbial forensic.

Nowadays, as microbial forensics is booming, an increasing number of scholars are beginning to explore this field. Through this review, it can clarify the fundamental concept of microbial forensics, showing the development trend and practical application throughout the whole development process of microbial forensics, so as to provide new ideas for subs.

## 2. Method

### 2.1. Database and search strategy

The Web of Science is one of the largest databases frequently used for retrieving relevant literature in different scientific fields. Among the databases, the Web of Science Core Collection is a collection of high-quality articles. In this study, the data related to the topic of microbial forensics were collected from the WoS Core Collection on 16 October 2022, which covers documents published from 1984 to 2022. The search strategy used in the database was as follows.

**Figure 1:**
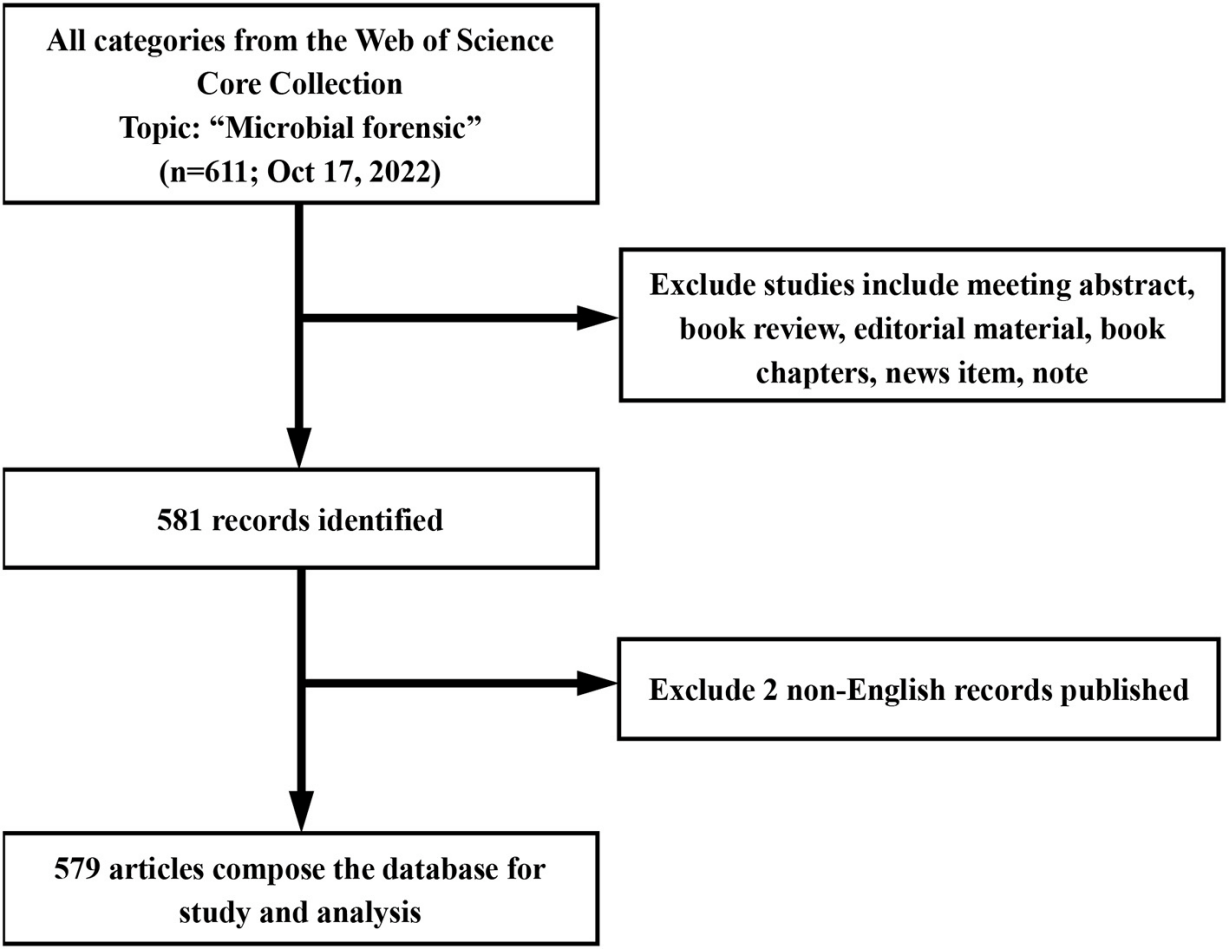
Search strategy.

First, the term “microbial forensics” was searched from all fields in the WoS Core Collection, and there were 611 records identified. Then, to improve the sensitivity and accuracy of the search results, all types of articles in the WoS Core Collection database were retrieved, excluding meeting abstract, book reviews, editorial material, book chapters, news item, and notes, leaving 581 records. Apart from that, from the dimension of language, the retrieval results at that time did not carry out language restrictions. Two non-English documents were excluded regarding the influence of English in the whole world applied to scientific communication, and finally, 579 records were obtained for further analysis. Then, the investigator would inspect the data of the records to make sure that the retrieved statistics could be included in this retrospective study. After the above screening, the eligible records were exported from the Web of Science and imported to the software [Biblioshiny (RStudio) and VOSviewer] to perform. In addition, the search strategy is presented in Figure 1.

### 2.2. Data analysis

To analyze the basic trend of the articles in microbial forensics, the following indicators were selected: the number of published articles per year, analysis of the 20 most published journals, analysis of the leading countries based on the most published documents, analysis of the 20 most published authors, analysis of the 20 most cited articles, analysis of the 20 co-occurrence keywords, and the trend topics. The statistics exported from the database were conducted by the Biblioshiny (RStudio), VOSviewer 1.6.18., and MS Excel to generate the visualized graphs, including the chronological distribution of publications, country co-occurrence map, the frequency and co-occurrence of the burst keywords, and the keywords time zone map, which could clearly show the features, trends, and frontiers of the field of microbial forensics. The visualized network map of country co-occurrence and the density map of the burst keywords co-occurrence were made with the help of VOSviewer. In the network visualization map, the thickness of the line between two countries could reflect the strength of the relationship based on the co-authorship. In the density map, every color system can depict one cluster, which could demonstrate the times of the keywords' co-occurrence. Finally, for both, the size of the circles or the areas can represent the number of the centrality about the indicators. The total citations (TC) are calculated from 1984 to 2022, using the Web of Science Core Collection. When the article is cited by itself or other articles, the number of citations is increased by one and then accumulated in turn. The h-index can be calculated by the following method. First, all the articles published by the scholar are ranked from high to low in terms of citation times and then searched from front to back until the citation times of an article are less than or equal to the article number; the article number obtained is the index which is as follows: *h*-index = max [min(*f* (*i*), *i*)], where “*f* (*i*)” represents the number of citations of the article, and “*i*” represents the serial number of the article.

## 3. Result

### 3.1. Chronological distribution of publications

**Figure 2:**
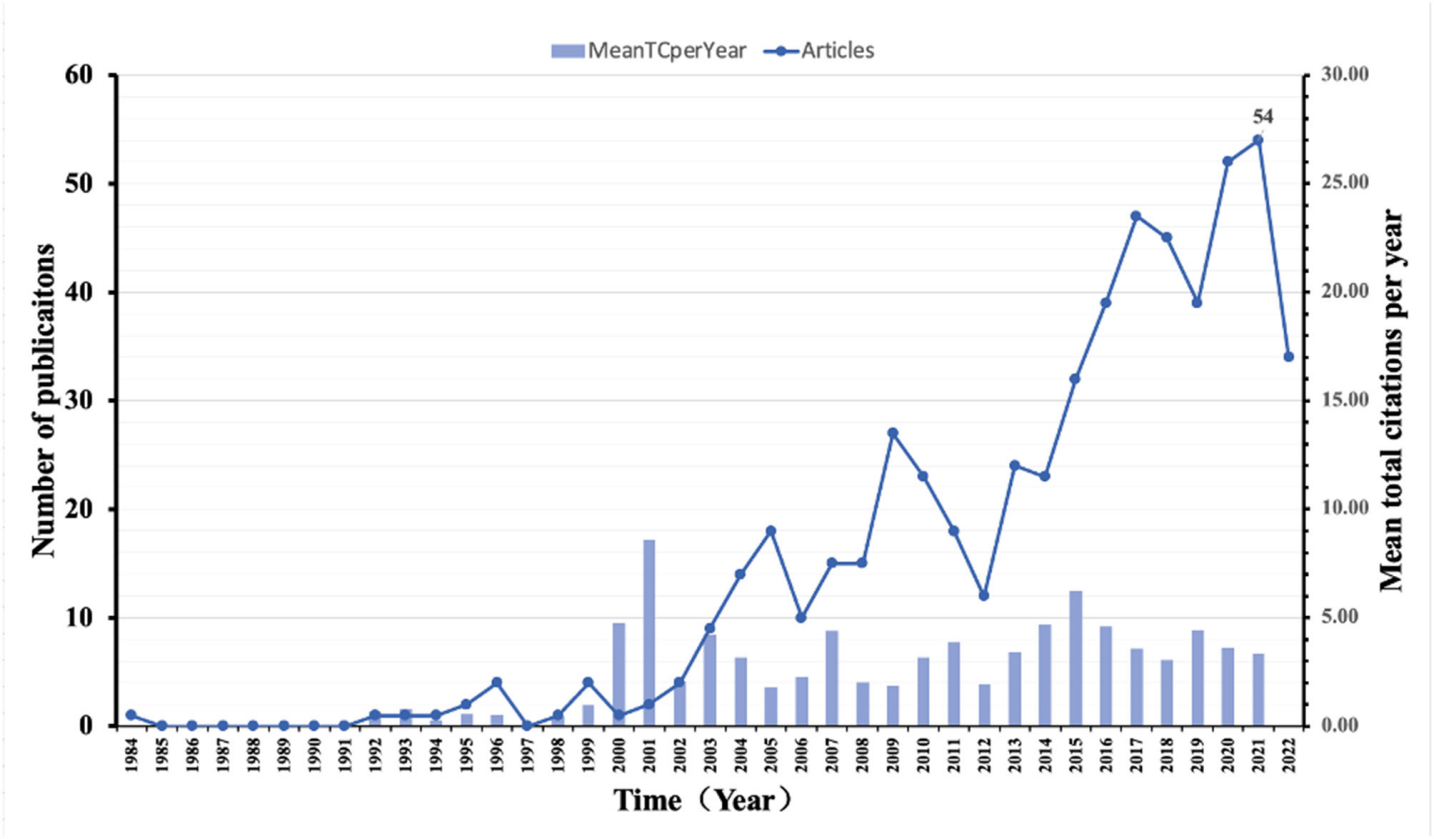
Chronological distribution of publications and mean TC per year in microbial forensics.

The chronological distribution of publications in microbial forensics is shown in Figure 2. The data revealed that the first research appeared in the year 1984, with only one publication. After a gap of 7 years, only one publication was contributed to the topic in 1992. Thereafter, the publications contributed to the topic are less than five in the 10 years. Until 2003, there was a slight increase in the number of publications, with nine publications being published. Thereafter, publications on the topic continued to be published regularly from 2003 to 2022. Moreover, the number of publications has gradually increased on the general trend, indicating the continuous development of the field of microbial forensics. The highest number of publications was contributed to 2021, with 54 publications this year.

The year 1984 had the lowest mean total citation (TC) per year with only 0.03. After a 7-year gap, the mean TC per year was up to 0.67 in 1992. In the next 7 years, the number of mean TC per year had been fluctuating below 1.00. In 2000, the number launched to 4.77 and has remained at a high level since then. The highest number appeared in 2001, with 8.60 per year, which suggests that the articles published in that year probably play an important role in this field.

### 3.2. Sources of publications

Table 1 highlights the top 20 most productive sources for publications on microbial forensics. The analysis ranked the “Journal of Forensic Sciences” at the top position in producing publications, followed by “Forensic Science International.” Regardless of articles, h-index or TC, the “Journal of Forensic Sciences” can rank No.1, which shows its leading role in this field. Moreover, from the PY-start, it is not difficult to learn that the “Journal of Forensic Sciences” is an old and well-established magazine on this topic. The remaining 18 sources were far behind the top two journals in terms of publications and total citations except “Forensic Science International Genetics.” Moreover, “Forensic in Microbiology” shows the potential for development obviously. With only 19 articles, it ranks fourth and has a relatively high h-index compared with other journals.

**Table 1:** Top 20 sources of publication in the field of microbial forensics (TC is short for total citation; PY_start is short for Publication years start).

**Journal (*n* = 579)**	**Number of articles**	**Percentage of total**	**h_index**	**TC**	**PY_start**
Journal of Forensic Sciences	51	8.81%	18	855	1992
Forensic Science International	47	8.12%	16	752	1996
Forensic Science International-Genetics	33	5.70%	15	559	2010
Frontiers in Microbiology	19	3.28%	8	256	2016
International Journal of Legal Medicine	18	3.11%	10	318	2005
PLoS One	18	3.11%	10	486	2010
Applied and Environmental Microbiology	15	2.59%	10	402	2002
Environmental Forensics	14	2.42%	6	181	2003
Journal of Forensic and Legal Medicine	12	2.07%	6	136	2010
Scientific Reports	11	1.90%	7	227	2016
Journal of Applied Microbiology	9	1.55%	5	114	
Microbiology Spectrum	9	1.55%	3	23	2016
Science & Justice	8	1.38%	6	119	2009
Journal of Microbiological Methods	7	1.21%	6	115	2008
Analytical and Bioanalytical Chemistry	6	1.04%	5	357	2004
Analytical Chemistry	5	0.86%	5	169	2003
Applied Microbiology and Biotechnology	5	0.86%	4	211	2011
Biosecurity and Bioterrorism-Biodefense Strategy Practice and Science	5	0.86%	3	37	2009
Criminal and Environmental Soil Forensics	5	0.86%	4	96	2009
Croatian Medical Journal	5	0.86%	4	188	1999

### 3.3. Leading countries

Among the research articles, for the multi-authored studies, the standard of the country to which the articles we count belong is the author's mailing address. The results show that the authors came from 40 countries, but there is one country whose name is not shown via bibliometric analysis. According to citations of the articles in 39 countries (Table 2), nine countries were ranked above the upper quartile, including the USA (*n* = 878 times), China (*n* = 126 times), Australia (*n* = 106 times), Italy (*n* = 81 times), France (*n* = 42 times), Germany (*n* = 40 times), Spain (*n* = 34 times), Sweden (*n* = 33 times), and the Netherlands (*n* = 31 times). The top three countries of the total citations received were the USA, Australia, and Canada. However, the average citations per article are led by Croatia, which ranks 37th in terms of the number of articles, while the United States ranks fifth.

**Table 2:** Top 39 countries contributed to microbial forensics (totally 40 countries contributed to the microbial forensics, but there is one country whose name is not shown via bibliometric analysis).

**Country**	**Number of articles**	**TC**	**Average article citations**
USA	878	8,088	29.52
China	126	437	10.40
Australia	106	930	26.57
Italy	81	163	9.59
France	42	51	7.29
Germany	40	596	45.85
Spain	34	254	18.14
Sweden	33	99	11.00
Netherlands	31	283	18.87
Switzerland	29	245	24.50
Canada	28	843	70.25
India	28	52	5.20
Japan	24	62	6.20
Poland	17	37	6.17
Turkey	16	27	6.75
New Zealand	15	203	29.00
Norway	14	80	20.00
Portugal	13	45	11.25
Israel	12	85	17.00
Romania	12	40	8.00
Brazil	8	54	27.00
Greece	7	107	26.75
Finland	7	9	4.50
Austria	6	9	9.00
Egypt	6	0	0.00
Iran	5	8	4.00
Saudi Arabia	5	4	4.00
Belgium	5	3	1.50
Czech Republic	4	14	4.67
Philippines	4	0	0.00
Denmark	3	74	37.00
Russia	3	8	2.67
Thailand	3	3	1.50
Argentina	2	2	2.00
Pakistan	2	2	2.00
Ireland	2	1	1.00
Croatia	1	138	138.00
United Kingdom		634	18.11
Korea		94	10.44

**Figure 3:**
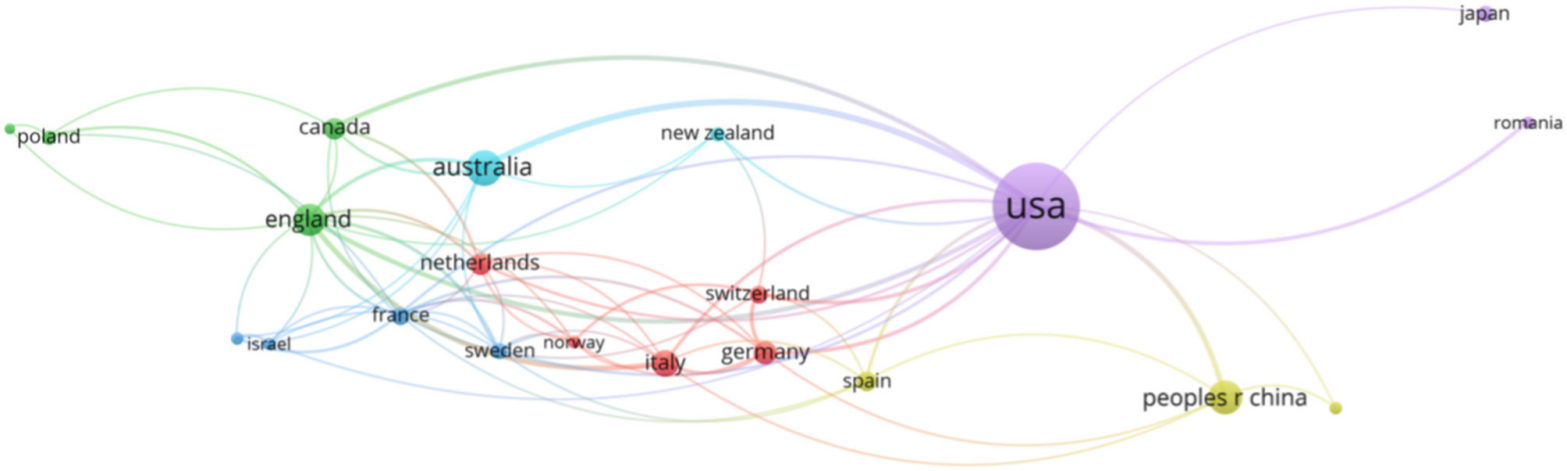
Co-occurrence of 21 countries.

After excluding the countries and regions that had no connection with others and limiting the minimum numbers (*n* = 5) of documents, 21 countries were analyzed in terms of the number of articles with VOSviewer (Figure 3). The countries' co-occurrence relation graph showed six clusters: (1) Italy, the Netherlands, Germany, Norway, and Switzerland; (2) Canada, England, Poland, and Portugal; (3) France, Sweden, Israel, and Scotland; (4) Spain, People's Republic of China, and Turkey; (5) USA, Japan, and Romania; and (6) Australia and New Zealand.

### 3.4. Most productive authors

Table 3 highlights the top 20 highly prolific authors in microbial forensics of the 478 first authors counted totally. The research productivity of the authors has been sorted primarily based on the number of articles, and for authors with the same number of publications, they were sorted once more based on the total citations received. The analysis ranked “Budowle B” at the top position with 22 articles published between 2004 and 2022, followed by “Carter DO” with 17 publications. The analysis ranked “Knight R” and “Keim P” at the sixth and seventh positions, whereas having the same number of articles, they possess a different number of citations. “KEIM P” was the first of the top 20 authors to start research in this field as well. Further analysis concerning the total citations of each author ranked “Carter DO” at the top position, despite being second in terms of the number of publications. In comparison, “Budowle B” ranked fourth based on the total citations received.

**Table 3:** Top 20 authors contributing to microbial forensics (“*n*” represents the total number of the first author in all articles).

**Rank**	**Author(s) (*n* = 478)**	**Number of articles**	**TC**	**First year**	**Last year**
1	Budowle B	22	575	2004	2022
2	Carter DO	17	1,257	2004	2021
3	Benbow ME	17	332	2015	2022
4	Tibbett M	12	802	2004	2013
5	Pechal JL	11	224	2015	2022
6	Knight R	9	1,214	2010	2021
7	Keim P	9	448	1999	2018
8	Finley SJ	9	299	2015	2022
9	Javan GT	9	299	2015	2022
10	Debruyn JM	7	273		
11	Fletcher J	7	186	2004	2014
12	Young JM	7	149	2014	2021
13	Jordan HR	7	100	2016	2020
14	Wunschel DS	7	77	2008	2018
15	Metcalf JL	6	575	2013	2021
16	Gilbert JA	6	379	2015	2020
17	Chakraborty R	6	208	2004	2008
18	Kreuzer-Martin HW	6	170	2003	2008
19	Schmidt CJ	6	78	2016	2020
20	Procopio N	6	42	2017	2022

### 3.5. Top 20 most cited publications

To identify the influential representative publications that contributed to microbial forensics research, Table 4 reveals the top 20 highly cited documents, with each >110 times. These records were almost published in authoritative journals and categorized into biological sciences, microbiology, analytical chemistry, etc. The analysis ranked “Darling AE, 2014, PEERJ” as the most influential publication with the highest number of total citations (377), followed by 371 and 360 total citations (TC) of “FIERER N, 2010, P Natl Acad Sci USA” and “Carter Do, 2007, Naturwissenschaften.” Especially, to deserve to be mentioned, the highest ranked document, “Darling AE, 2014, PEERJ,” also ranks first in securing total citations per year (TCPY), followed by “Metcalf JL, 2016, SCIENCE.”

**Table 4:** Top 20 most cited publications in the field of microbial forensics (CY is short for citation years; TCPY is short for total citations per year).

**Article title**	**Journal**	**TC**	**CY**	**TCPY**
Phylosift: phylogenetic analysis of genomes and metagenomes	PEERJ	377	9	41.89
Forensic identification using skin bacterial communities	Proceedings of the National Academy of Sciences of the United States of America	371	13	28.54
Cadaver decomposition in terrestrial ecosystems	Naturwissenschaften	360	16	22.50
Development of oil hydrocarbon fingerprinting and identification techniques	Marine Pollution Bulletin	351	20	17.55
Compound-specific stable isotope analysis of organic contaminants in natural environments: a critical review of the state of the art, prospects, and future challenges	Analytical and Bioanalytical Chemistry	294	19	15.47
Supervised classification of human microbiota	FEMS Microbiology Reviews	263	12	21.92
Microbial community assembly and metabolic function during mammalian corpse decomposition	Science	255	7	36.43
Identifying personal microbiomes using metagenomic codes	Proceedings of the National Academy of Sciences of the United States of America	247	8	30.88
Anthrax molecular epidemiology and forensics: using the appropriate marker for different evolutionary scales	Infection Genetics and Evolution	246	19	12.95
Determination of nitroaromatic, nitramine, and nitrate ester explosives in soil by gas chromatography and an electron capture detector	Talanta	223	22	10.14
A microbial clock provides an accurate estimate of the postmortem interval in a mouse model system	Elife	182	10	18.20
Electron mediators accelerate the microbiologically influenced corrosion of 304 stainless steel by the desulfovibrio vulgaris biofilm	Bioelectrochemistry	180	8	22.50
Stable isotope ratios of tap water in the contiguous united states	Water Resources Research	155	16	9.69
DNA typing from skeletal remains: evaluation of multiplex and megaplex Str systems on DNA isolated from bone and teeth samples	Croatian Medical Journal	138	22	6.27
The living dead: bacterial community structure of a cadaver at the onset and end of the bloat stage of decomposition	PLoS One	124	10	12.40
Plant pathogen forensics: capabilities, needs, and recommendations	Microbiology and Molecular Biology Reviews	115	17	6.76
Whole-genome sequencing in outbreak analysis	Clinical Microbiology Reviews	115	8	14.38
Population genetic analysis infers migration pathways of phytophthora ramorum in us nurseries	Plos Pathogens	113	14	8.07
A comprehensive evaluation of multicategory classification methods for microbiomic data	Microbiome	112	10	11.20
A survey of the methods for the characterization of microbial consortia and communities	Canadian Journal of Microbiology	112	18	6.22

### 3.6. Top keywords and co-occurrence network on microbial forensics

The top 20 keywords, retrieved from the keywords plus the 579 documents based on the frequency of occurrence, are presented in Table 5. The total occurrences of keywords were 3,924. Among the top 20 keywords, with a minimum occurrence of 18, diversity, identification, DNA, and bacteria appeared to be the most prominent keywords on the list. “Diversity” was the most important term with the highest occurrences of 101 (2.57%), followed by 91 and 45 frequencies (2.32%; 1.15%) of “identification” and “DNA,” respectively.

**Table 5:** Top 20 keywords in microbial forensics.

**Keywords**	**Cluster**	**Links**	**Total link strength**	**Occurrences (*n* = 3,924)**	**Percentage of total**
Diversity	3	78	328	101	2.57%
Identification	3	81	287	91	2.32%
DNA	1	64	145	45	1.15%
Bacteria	3	57	136	41	1.04%
Decomposition	2	41	121	38	0.97%
Time	2	46	138	38	0.97%
Bacterial	3	44	89	28	0.71%
Succession	2	43	100	28	0.71%
Microbial forensics	1	28	54	27	0.69%
Potential use	2	33	100	26	0.66%
Soil	2	48	94	25	0.64%
Bacillus-anthracis	1	34	82	23	0.59%
PCR	4	38	67	23	0.59%
Death	2	36	92	22	0.56%
Microbial degradation	5	11	27	21	0.54%
Samples	3	35	51	21	0.54%
Community	3	37	80	20	0.51%
Extraction	4	39	62	20	0.51%
Sequences	3	42	77	20	0.51%
Evolution	1	28	51	18	0.46%

**Figure 4:**
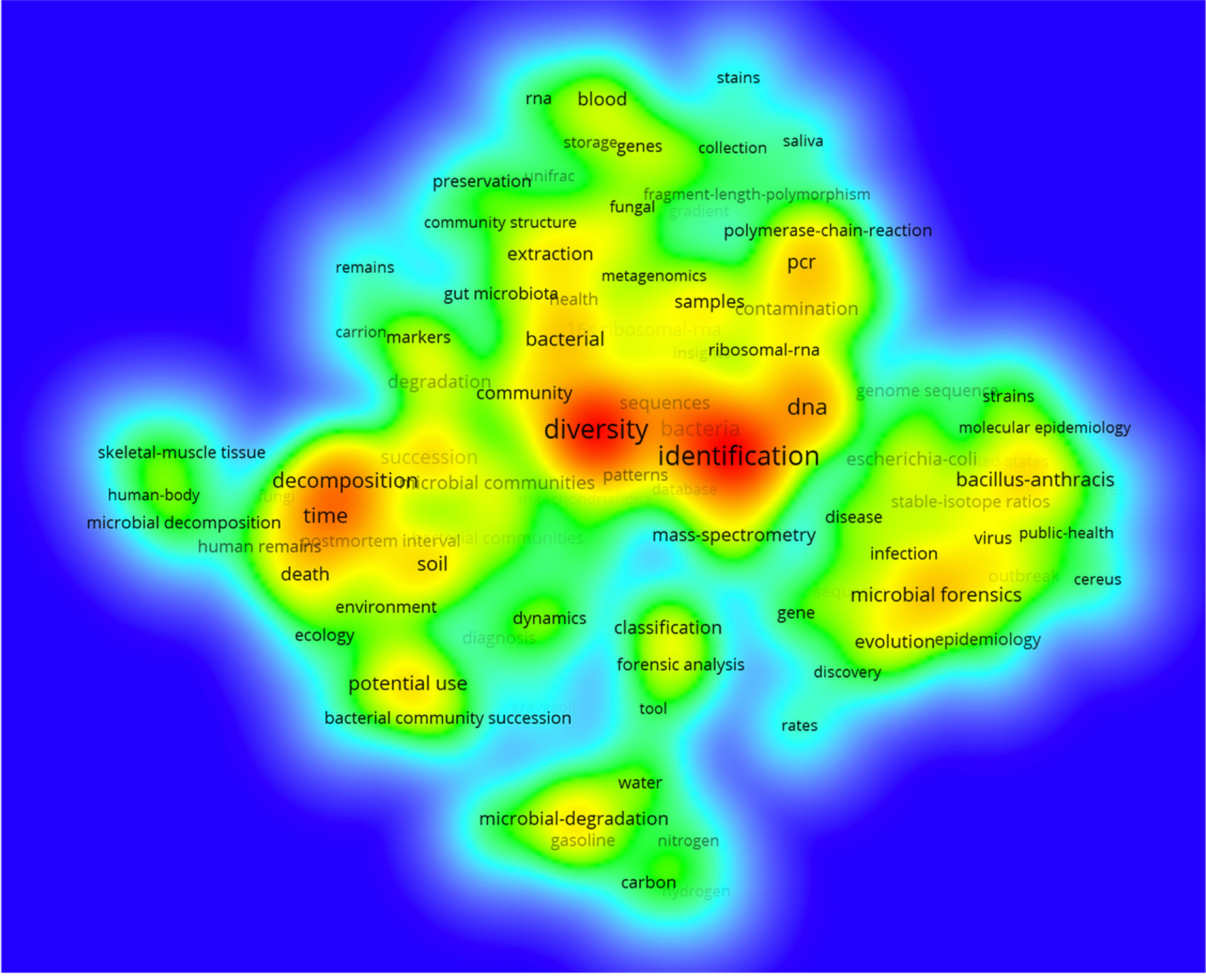
Co-occurrence terms on microbial forensics. The density reflected the co-occurrence times of terms.

The co-occurrences of keywords are shown in Figure 4. To analyze and visualize the network of keywords co-occurrence, VOSviewer 1.6.18 has been used to create a density visualization map including 100 terms, illustrating the degree of the frequency and relevance of the keywords. The analysis showed that the 100 terms can be classified into six groups, which include 25, 23, 23, 15, 12, and 2 keywords, respectively. Interestingly, the clusters of the top 20 keywords are focused on Clusters 2 and 3 (Table 5). Among 100 statistics, although “diversity” ranked first in the co-occurrence total link strength (total link strength = 328) and the frequency, the links of “identification” surpassed that of “diversity” with the number 81.

### 3.7. Trend topics

**Figure 5:**
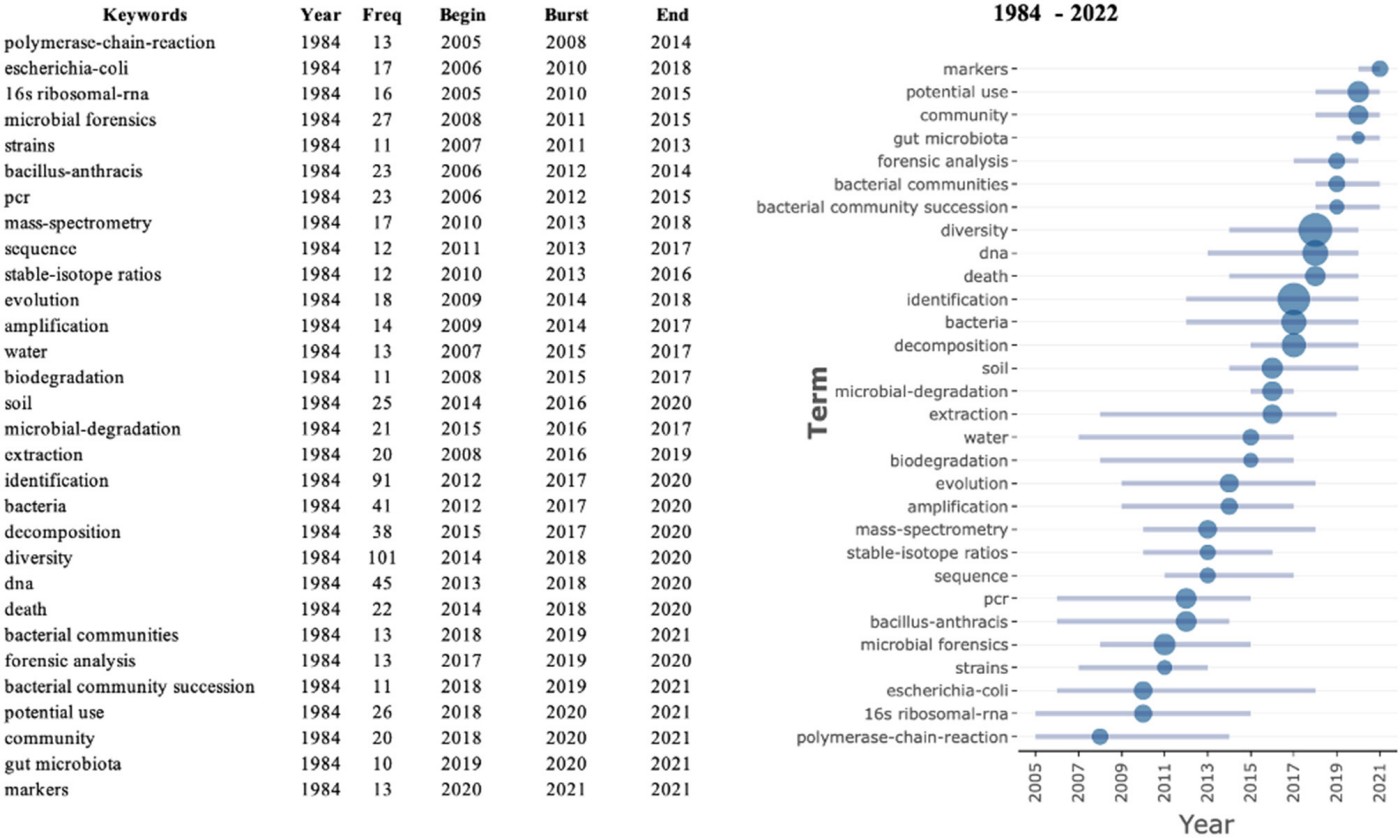
Top 30 keywords with strongest citation bursts. The figure has taken on the change for years from 1984 to 2022. While the blue line indicates the years in which keywords appeared, the blue point represents the burst year of the keywords (the larger the dot, the greater the strength).

To obtain the statistics of keywords with strong bursts in scientific research, Figure 5 displays the change of trend topics for years from 1984 to 2022. The graph presents the hot spot issues by showing the strength (frequency) and time duration. Among the top 30 keywords, the early research began to concentrate on the “polymerase-chain-reaction” and “16s ribosomal-RNA,” which emerged as keywords in 2005 and became trend topics with the frequency of 13 and 16, respectively, till 2008 and 2010. Then, as microbial forensics progressed, new trend topics gradually appeared and transferred. “Diversity,” ranked first with a frequency of 101, started to gain popularity in 2014 and reached its peak in 2018. The second strongest word was “identification” with a frequency of 91. Emerging from 2012 to 2020, “identification” burst in 2017 before that of “diversity”, although it was in the second position generally.

## 4. Discussion

### 4.1. The origin of the discipline of microbial forensics

The birth of microbial forensics originated from the combination of two longstanding disciplines—forensic medicine and microbiology. The history of forensic medicine can be traced back to the Code of Hammurabi, King of Babylon, ~2,200 before Christ (Davis, 1985). After that, forensic medicine went through a slow stage of development and moved toward modernization with the continuous development and improvement of the legal system and research technology in the early 20th century. Microbiology owes its beginnings to Robert Hook, a British scientist who created a compound microscope in 1665, allowing him to observe the cellular structure for the first time. Modern medical microbiology emerged through the pioneering work of Louis Pasteur and Robert Koch approximately 150 years ago (Doolittle et al., 2018). With the advent of molecular biology technologies such as polymerase chain reaction, real-time fluorescent quantitative PCR, and DNA sequencing in the 1980s, and the rapid development of metagenome sequencing technology at the turn of the 21st century, microbiome research was born. The rapid progress in microbial sequencing and bioinformatics technology has greatly improved the technical level of microbial identification and promoted the emergence of microbial forensics medicine.

The early collaboration between the two disciplines can be traced back to the emergence of soil forensics in the mid-19th century as part of crime scene investigation, aimed at locating suspects and solving crimes (Santiago-Rodriguez and Cano, 2016). Due to the complexity of soil, which includes diverse microorganisms, it is considered one of the most intricate ecosystems on earth, providing the foundation for the credibility of soil microbial forensics (Ruffell, 2010). In the history of forensic genetics, the role of soil microbial forensics is undoubtedly a milestone. However, due to the limitation of the Sanger sequencing approach, the number of articles published in related fields was low in the subsequent years. The situation took a favorable turn in 2001—the *Bacillus anthracis* envelope attack after the “9.11” terrorist incident in the United States (Keim et al., 2008). The attack marked the first formal cooperation between forensic science and microbiology. It was also in response to this that forensic microbiology was first proposed as a discipline in 2001. Dr. Budowle, the then-director of the FBI's forensic DNA laboratory, defined it as “analyzing evidence from a bioterrorism act, biocrime, or inadvertent microorganism/toxin release for attribution purposes.” This may explain why the field of microbial forensics had the largest number of the lowest total citation (TC) per year in 2001.

As the pioneer to propose forensic microbiology as a discipline, the United States has been a leader in this field. This is consistent with previous statistics that the American authors have produced the most publications, and the country itself has produced the highest number of publications, surpassing those of other countries. The reasons for this dominance are easily understood. The United States boasted significant resources to invest in advanced scientific and technological research and to offer adequate resources and incentives to researchers (Zangpo et al., 2022), which greatly facilitated the development and innovation of microbiological research techniques. Moreover, technological innovation has further enabled the development of microbial forensics. More crucial than these objective conditions was the government's recognition of the importance of research in microbial forensics. In the October 2001 incident in the United States, anthrax spores were sent by mail, resulting in the development of inhalation anthrax that caused the death of five people and infected 17 others (World, 2004). This case generated a great deal of governmental concern and challenged the US public health infrastructure, which was also strained by the continued emergence of new microorganisms such as Hantavirus, Ebola, and *Escherichia coli* strains (Bryan and Fields, 1999). In response, the US decided to vigorously support its public health infrastructure. Unrestricted funding and heightened focus in this area have produced many well-trained researchers and provided the latest research equipment, contributing greatly to the advancement of microbial forensics.

Over the last 20 years, the field of microbiological forensics has undergone significant growth, expanding from being limited to the detection of pathogenic microorganisms to encompassing a range of detection techniques with diverse applications. These techniques now include the time of death assessment (DeBruyn and Hauther, 2017), individual identification (Franzosa et al., 2015), determination of the place or manner of death (Lee et al., 2017), and the potential to detect difficult cases. As a result, microbiological forensics has consequently emerged as a discipline that employs scientific methods to analyze microbial evidence for investigative purposes in both criminal and civil cases (Schmedes et al., 2016). The expansion in research has also led to a gradual increase in the number of studies on microbial forensics from 2003 to 2022.

### 4.2. Main focus of research in microbial forensics

Through the analysis of keywords and the trend of topics, it has been observed that scholars are increasingly focusing on studying the characteristics of microorganisms such as microbial diversity. Such features can help scholars better integrate the study of microorganisms into the practical applications of forensic medicine.

In terms of microbial diversity, it means the differences in the composition and abundance of microbial communities within human microecosystems, which serve as the basis for utilizing microorganisms in forensic genetics research, especially identification. With the in-depth study of microorganisms, it has become increasingly clear that the species diversity and genetic diversity of human microbiota are crucial factors in subsequent analysis and application in forensic practice, and the data have shown that the researchers attached great importance to diversity for a long time in the field of microbial forensics (Li and Zhang, 2012; Tierney et al., 2019). Notably, there were significant differences in the composition of microbial communities across different parts of the human body, among which the skin, oral cavity, and intestinal tract have the most characteristic microbial communities (Costello et al., 2009; Chen and Tsao, 2013). Furthermore, microbial communities in specific regions of the body also exhibit individual specificity, which is closely linked to life-history parameters, including genetics, age, diet, health status, and environment (Bik et al., 2010; Ding and Schloss, 2014). It enables microbes to have the potential to be employed in body fluid identification and individual identification.

In addition, there is a term, “thanatomicrobiome,” which refers to the study of the microorganisms, found in internal organs and cavities upon death derived from the Greek word “Thanatos,” meaning death (Javan et al., 2016). Some studies have revealed that the microbial community changes accordingly within a certain period after death, and microbial diversity also varies with the time of death. This highlights the potential role of microorganisms in estimating postmortem interval (PMI) in forensic pathology. The traditional method of PMI estimation relied heavily on subjective observation of cadaveric temperature, ecchymosis, and stiffness degree (Kaatsch et al., 1993; Muggenthaler et al., 2012; Brooks, 2016), but this method was limited by their reliance on experience. Under this circumstance, forensic entomology can provide insights by identifying the species and geographical distribution of insects, determining the growth and development state, and estimating PMI by combining the community succession law of insects on the corpse through the inspection, identification, and classification of insect specimens collected from the environment inside, outside, or near the corpse. Moreover, there are still some limitations, such as their unavailability in different seasons, different timing of oviposition, and variation in development (Hilal et al., 2021). However, because of its ubiquitous nature and predictable ecologies, microbes can be used as physical evidence in forensics. Moreover, there are many microbes in a human body, originally, which are called microbiome (Zhou and Bian, 2018). Based on the animal experiments that have been conducted (e.g., pig, cow, mince, and human), it has been found that the species and abundance of microbe will change with time (Metcalf et al., 2013; Mason et al., 2023). However, it is feasible to infer the time of death based on changes in the microbiome after death. Then, to infer the time of death, it is necessary to examine the dynamics of the microbial community in the internal organs of the human body, as well as that associated with the outside of the human corpse, referred to as the surface decay microbiota (Dash and Das, 2020). Their succession changes regularly with the decomposition of corpses; thus, the dynamic analysis of their communities can provide an effective basis for estimating the time of death (Metcalf et al., 2016).

In addition to the role of postmortem microbial communities in estimating PMI, the use of changes in microbial community composition and structure induced by decomposition of corpses can also be leveraged to explore the processes of production and transmission of high-risk ARGs to inform environmental governance and public health. As hosts of ARGs, microorganisms usually exist in the animal microbiome. As the corpse decomposes, these bacterial communities carrying ARGs may transfer from the skin, gastrointestinal tract, and other systems of the corpse to environmental media, which affects the natural occurrence and distribution of ARGs in the environment and increases the abundance and diversity of ARGs in bulk water (Feng et al., 2021). Moreover, given that the diversity of microbial communities is impacted by precipitation, rainfall would induce a gradual differentiation of bacterial communities in the soil, where the corpses are decomposed, significantly changing the abundance and community structure of bacteria. It has been seen that the moderate rainfall group displays a faster differentiation rate than the heavy rainfall group (Su et al., 2022). Similar to precipitation, microbial community changes are also affected by the amount of water. Therefore, by regulating water quantity, the “dilution effect” can be realized to a certain extent, offsetting or weakening the influence of the composition and structure of the microbial community as well as the spread and distribution of ARGs in environmental media caused by the decomposition of corpses (Yang et al., 2021).

Furthermore, it is insufficient to solely focus on microbial community succession during cadaver decomposition. The study of microbial metabolites and their correlation with the microbiota can also play a crucial role in forensic pathology. For example, the decomposition of corpses requires the enrichment of some bacteria to perform, while the growth and reproduction of bacteria require amino acids to synthesize proteins. As a result, amino acid metabolites are enriched in the corresponding environmental media of corpses. At the same time, metabolites produced by microorganisms from decomposing endogenous and exogenous components in soil can also serve as pertinent indicators in forensic science and other scenarios (Zala, 2007; Alan and Sarah, 2012; Santiago-Rodriguez et al., 2013; Finley et al., 2015; Santiago-Rodriguez and Cano, 2016).

When studying the succession of microbial communities during the decomposition process, many scholars initially employed real-time fluorescence quantitative PCR technology. However, in the actual case, the environment, where the corpse is located, is complex, and the spoilage flora is extremely diverse, so it is difficult to explain the overall change of the flora only by understanding the abundance change of one or a few bacteria species. As sequencing technology continues to develop and mature, high-throughput sequencing (HTS) has emerged as a vital tool for identifying microbial species and abundance (Kakizaki et al., 2012; Hyde et al., 2013; Carter et al., 2015).

In brief, the intersection of microbiology with forensic genetics and forensic pathology is a current area of intense research interest. This cross-disciplinary field also encompasses environmental governance and public health concerns related to corpse decomposition, which also suggests some problems that microbial forensics medicine endeavors to address.

### 4.3. Advances in technology led to a boom in the field of microbial forensics

With the advancement of science, some molecular biology technologies have experienced rapid development. Notably, the research of polymerase chain reaction (PCR) technology burst onto the scene in 2008, slightly earlier than the time when microbial forensics medicine became a trending topic. This chronological correlation further proves that PCR technology has promoted the field of microbial forensics. The advent of PCR is undoubtedly a watershed moment in the history of microbiology, as it greatly facilitated subsequent gene expression studies through amplifying, modifying, and cloning genes (Canene-Adams, 2013). For example, some scholars used PCR to amplify DNA extracted from the soil with universal eubacterial primers, thereby increasing the number of target genes to facilitate subsequent sequencing (Heath and Saunders, 2006). The innovative PCR method provides necessary technical support for the sustainable development of microbial forensics in the 21st century. Apart from that, there are statistics showing that the emergence of “sequencing” coincided with the boom of the next-generation sequencing (NGS, starting in 2005) in approximately 2011 (Liu et al., 2012; Børsting and Morling, 2015). With the rapid development of the NGS, scholars can gain profound insights into microorganisms through a faster and more economical way, which is serviced for the research of microbial forensics (Suárez Moya, 2017). For instance, investigating microorganisms by using NGS can aid in identifying the cause and the time of death (Cox et al., 2013; Finley et al., 2015). Additionally, sequencing the microbiome in various habitats, such as the skin, the saliva, and soil, demonstrated the similarities and differences among individuals and locations (Giampaoli et al., 2012, 2014; Sucher et al., 2012). The development of PCR and the maturation of NGS technology have promoted the research of scholars on microbial forensics.

As researchers delved further into this field, they gradually identified some potential applications of microorganisms. In recent years, several studies have emerged to verify the potential of microorganisms in forensic practice. For example, some scholars assessed the method of narrowing down the search area and determining the origin of samples via microorganisms in the soil (Demanèche et al., 2017; Habtom et al., 2019). Some types of research analyzed the decomposition of microorganisms to estimate the PMI (Gouello et al., 2021). In addition, certain special samples have become the focus of scholars in their pursuit of breakthrough in the field of microbial forensics. Initially, researchers mostly used microbes in the soil and skin to do their research. Gut microbiota, as one of the special samples, was gradually incorporated into the research of microbial forensics. Studies have demonstrated that gut microbiota can be used to reflect age-related and sex-related changes, helping to roughly identify sexes and different age groups (de la Cuesta-Zuluaga et al., 2019; Takagi et al., 2019; Huang et al., 2020; He et al., 2021). Moreover, some studies also found that gut microbiota held the potential for individual identification (Wang et al., 2022). Moreover, in recent years, the research direction in the field of microbial forensics started to use “marker” as an effective tool. Given the huge number of microorganisms existing in the human body, which could be specified in different individuals, microbial markers offered a promising approach in the field of microbial forensics (Sijen, 2015). In the latest study, microbial markers have even been employed to identify biological traces of microorganisms at crime scenes and ultimately promoted personal identification (Salzmann et al., 2021). Beyond that, some studies have also taken into account the influence of microbial associations on different parts of the human body, carried out relevant cross-over studies, and classified the resulting microbial markers, which demonstrate a high body part classification ability and are hopeful to be applied to high-precision identification tasks in forensic practice (Tackmann et al., 2018).

## 4. Conclusion

This study focused on the trend and research orientations of microbial forensics. From the overall trend, global research in the field of microbial forensics has gradually gained attention, with an increasing number of publications over the years. At the national level, the relative research is mainly driven by developed and developing countries, such as the USA and China, but it is hoped that more countries will engage in this field in future. At the same time, it was found that although the field of microbial forensics was relatively new and small compared with other branches of forensic medicine, it is closely linked to the practical application of many important forensic fields.

## Author contributions

FS and HL conceptualized the study. XG, LG, and YL analyzed the statistics and wrote the manuscript. XG and SW edited the manuscript. All authors contributed to the article and approved the submitted version.
